# Development of reconfigurable smart medical wards using integrated components and complex features

**DOI:** 10.3389/fdgth.2026.1784460

**Published:** 2026-05-15

**Authors:** Ramesh Kumar Veerapaneni, Radhakrishnan Delhibabu, Dmitriy Levonevskiy, Nataly Zhukova

**Affiliations:** 1 School of Computer Science and Engineering, Vellore Institute of Technology, Vellore, Tamil Nadu, India; 2 Laboratory of Big Data Technologies in Socio-Cyberphysical Systems, St. Petersburg Institute for Informatics and Automation of the Russian Academy of Sciences, St. Petersburg Federal Research Center of the Russian Academy of Sciences (SPC RAS), Saint Petersburg, Russia

**Keywords:** applications in medicine, automation of medicine, complex features, disease diagnostics, smart ward

## Abstract

Patient treatment in hospitals requires their regular monitoring to assess their health conditions. At the same time, routine measurements are often delayed, missed, or not analyzed, and this situation worsens at night and on weekends due to reduced staffing, which in turn affects the quality of treatment. Innovations in the field of infocommunication technologies allow the healthcare industry to enhance the provided medical services. One of the promising areas is the creation of smart wards so that the patient information will be easily accessible and properly analyzed, with real-time alerts sent to healthcare professionals. Assessing health and diagnosing diseases require data collection and processing techniques that can be implemented by the smart ward modules. The variety of data, situations, diseases, and techniques creates the need to simplify and speed up the development of such modules and to increase the reusability of such development. In this work, we propose a method that allows the creation of applications as integrated components for smart wards for assessing patient health using complex feature techniques. The proposed method provides the necessary software flexibility and reusability for smart wards, enabling dynamic construction of processing pipelines. Thus, it becomes possible to reduce labor costs not only in the application usage but also in the development of such components.

## Graphical Abstract

**Figure:**
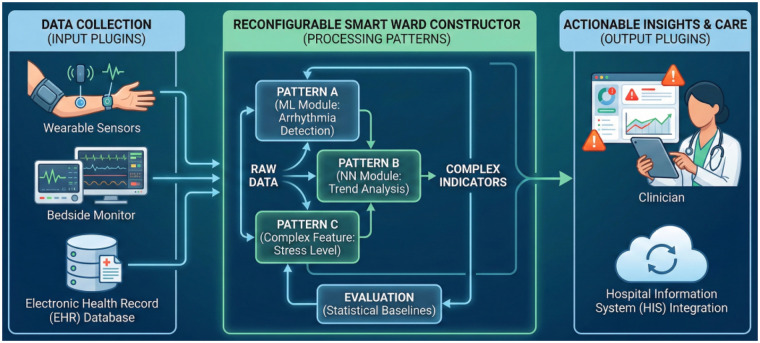

## 1 Introduction

Smart medical wards, representing a convergence of Medical Cyber-Physical Systems (MCPS) and the Internet of Medical Things (IoMT), have emerged as a critical solution for modernizing inpatient care. These environments are designed to transform traditional hospital rooms into intelligent ecosystems capable of autonomous data acquisition, real-time analysis, and decision support. By integrating advanced sensors, wearable devices, and Electronic Health Records (EHR) into a unified network, smart wards aim to automate patient care processes, enhance safety protocols, and optimize clinical workflows. This technological shift is essential for addressing systemic challenges in healthcare, such as staff shortages, alarm fatigue, and the increasing complexity of patient conditions in aging populations. Recent advancements in smart hospital architectures, automated patient monitoring, and AI-driven diagnostic systems have been extensively documented in the recent literature (1–22).

The economic and operational imperatives for adopting these technologies are substantial. The global smart hospital market, valued at approximately USD 63 billion in 2024, is projected to expand to over USD 390 billion by 2035, driven by a Compound Annual Growth Rate (CAGR) of 17%–20% (23). This growth trajectory underscores the industry’s reliance on remote patient monitoring (RPM) and automated analytics. Clinical studies corroborate the efficacy of these systems; for instance, automated monitoring has been shown to reduce the time nursing staff spend on routine vital sign checks by over 50%, thereby increasing the time available for direct patient interaction by nearly 43% (24). Furthermore, continuous surveillance combined with intelligent data processing has demonstrated the potential to reduce hospital-acquired infections by up to 40% and lower readmission rates through the early detection of physiological deterioration (25).

Despite these advancements, a significant bottleneck remains in the *configurability* and *interpretability* of the data generated. Modern smart wards generate massive volumes of heterogeneous data—ranging from simple scalar values like temperature to complex time-series data like ECG waveforms. Traditional monitoring systems typically rely on simple threshold-based logic (e.g., triggering an alarm if heart rate exceeds 100 bpm). However, such linear approaches are often insufficient for early diagnosis and personalized care, as they fail to account for the patient’s context, medication history, or circadian rhythms. This rigidity often results in a high rate of false positives, contributing to alarm fatigue among medical staff.

To address these limitations, we propose a shift from simple metrics to **Complex Features**. A Complex Feature is not merely a raw data point but an integrated indicator constructed using a combination of Machine Learning (ML), Neural Networks (NN), and statistical analysis. Unlike simple raw metrics, a complex feature aggregates multiple data streams to represent a higher-level health state (e.g., “Stress Level” derived from HRV and Galvanic Skin Response, rather than just “Heart Rate”). Crucially, the estimation of these values is not static. The determination of whether a complex feature is “normal” or “pathological” must be dynamic, depending on the individual peculiarities of the patient and their specific disease progression. This capability is fundamental to realizing the promise of personalized medicine within a smart ward environment.

Implementing this approach poses a software engineering challenge: how to build systems that allow medical professionals to define these complex features without needing to rewrite the underlying code for every new patient or condition. We view the data processing procedure as a “constructor” or a modular framework. In this model, modules with specific logic—such as a specific NN architecture for arrhythmia detection or a regression model for trend analysis—are assembled into processing pipelines on the fly using predefined rules. This architecture ensures that the smart ward system remains flexible, reusable, and scalable.

### 1.1 Contributions

The article discusses the current state of the art in the field of smart wards and smart technologies in medicine and proposes a novel approach to their design. The main contributions of this work are conceptually divided into theoretical and methodological advancements:

- **Theoretical contribution:** The formalization of a mathematical and conceptual model (M=⟨I,P,V,E,T⟩) for patient health assessment in medical wards. This model abstracts raw data streams into higher-level representations using “Complex Features,” fundamentally shifting evaluation logic from static scoring to dynamic, context-aware physiological baselining.
- **Methodological contribution:** The development of a reconfigurable “Constructor” method that treats data processing algorithms as interchangeable “Processing Patterns.” This decouples clinical logic from hardware dependencies, enabling the flexible and dynamic assembly of data processing pipelines on the fly without requiring software engineering intervention.
- **Experimental validation:** The practical validation of the proposed method using simulation data for various medical scales (PSHA, Glasgow Coma Scale, Grace, and Apanasenko), demonstrating significant reductions in assessment latency and improvements in diagnostic accuracy.

The remainder of this paper is organized as follows: Section 2 reviews the related work in the field of smart wards, IoT middleware, and existing health data platforms. Section 3 formulates the research problem, defining the limitations of current scoring systems and the necessity for complex indicators. Section 4 details the proposed “constructor” method, formally defining the model inputs, processing patterns, and evaluation criteria. Section 5 presents the validation of the method, comparing the efficiency and accuracy of the proposed approach against manual and semi-automated baselines. Finally, Section 6 concludes the paper and discusses future directions for research in medical automation.

## 2 Related work

The development of smart wards and medical cyber-physical systems (MCPS) has evolved significantly in recent years. This evolution has transitioned from simple, localized data collection to complex, ecosystem-wide architectures capable of AI-driven analysis and decision support. The current state of the art can be analyzed through three distinct dimensions: architectural evolution, commercial platform capabilities, and data processing paradigms.

Numerous studies have focused on the implementation of IoT and edge computing in smart hospital environments (1–5). Furthermore, the integration of automated data processing for patient monitoring—specifically addressing paroxysmal sympathetic hyperactivity and similar syndromes—has seen significant development (6–20). Finally, cloud infrastructure and advanced AI algorithmic paradigms have been proposed to support these heavy computational loads securely (11–22).

### 2.1 Evolution of smart ward architectures

Early iterations of smart wards focused primarily on the digitization of paper records and basic telemetry. However, recent studies from 2023–2025 highlight a paradigm shift towards integrated, “hospital-at-home” and hybrid architectures. For instance, Cheng et al. (26) proposed a model emphasizing the seamless continuum of care between hospital and home environments, relying heavily on ambient sensing (26).

A critical debate in current literature concerns the balance between **Cloud Computing** and **Edge Computing**. While cloud platforms offer unlimited storage and processing power for retrospective analysis, they suffer from latency issues critical in emergency scenarios. Abdellatif et al. (27) highlighted the necessity of edge-based compression and classification to ensure real-time responsiveness in smart healthcare systems (27). Modern smart wards are increasingly adopting a hybrid approach, where immediate “reflex” actions (e.g., fall detection) occur at the edge, while “reflective” analysis (e.g., long-term trend prediction) occurs in the cloud.

### 2.2 Commercial and industrial platforms

To provide context for the proposed “constructor” method, it is essential to analyze existing industrial platforms. These solutions generally fall into two categories: hyperscale cloud providers offering healthcare APIs, and specialized medical device middleware.

#### 2.2.1 Cloud-based health data platforms

Major providers have launched HIPAA-eligible services designed to aggregate health data. AWS HealthLake (28) and Google Cloud Healthcare Data Engine (29) utilize the FHIR (Fast Healthcare Interoperability Resources) standard to normalize data from disparate sources. These platforms excel at large-scale analytics and interoperability but often lack the granular, reconfigurable logic required for specific bedside monitoring tasks.

#### 2.2.2 Device integration and IoT middleware

Platforms like Validic (30) and Capsule Technologies (31) focus on the “last meter” of connectivity, ensuring data correctness from medical devices before it reaches the Hospital Information System (HIS). Open-source alternatives like ThingsBoard (32) are frequently used in research for rapid prototyping due to their customizable rule chains.

Table 1 presents a comparative analysis of these platforms, highlighting the trade-off between standardization and reconfigurability.

**Table 1:** Comparative analysis of existing IoT and health data platforms.

Platform/solution	Primary focus	Key strengths	Limitations for smart wards
AWS HealthLake/Google cloud	Data aggregation	Scalability, FHIR native support, integration with massive AI models.	High latency (Cloud-only); rigid for defining custom, patient-specific real-time logic.
Capsule tech/Validic	Device connectivity	Vendor-neutral device drivers, high data fidelity, clinical validation.	Functions primarily as a “pipe”; limited capability for complex, multi-variable feature generation.
ThingsBoard (Open Source)	IoT management	Flexible rule chains, visualization, widely used in prototyping.	Generic IoT focus; lacks specific medical data structures and clinical safety layers out-of-the-box.
TactioRPM	Remote monitoring	Patient engagement, mobile app integration, chronic care workflows.	Focuses on out-patient settings; less suited for high-frequency, intensive inpatient ward monitoring.

### 2.3 Data processing paradigms

In the domain of data processing, the focus has shifted from rule-based systems to multimodal deep learning. Alloh et al. (33) reviewed machine learning approaches in smart healthcare, noting a trend towards “spontaneous intelligence,” where systems predict patient deterioration using heterogeneous IoT data streams (33). The necessity for robust, generalized AI architectures extends beyond inpatient wards; recent studies have demonstrated the transformative impact of integrated AI models across various domains (22), emphasizing the need for modularity when deploying predictive analytics in complex clinical settings. Furthermore, advancements in Large Language Models (LLMs) like Med-PaLM have opened avenues for interpreting unstructured medical data (34), though their real-time integration remains experimental.

Despite these advances, a significant gap exists in how these processing rules are defined and deployed. Most current systems operate on a *Static AI* model—where a model is trained, frozen, and deployed. They lack the flexibility to “construct” new diagnostic pathways on the fly without software engineering intervention.

Table 2 illustrates the evolution of these processing paradigms and positions the proposed “Complex Features Constructor” within this landscape.

**Table 2:** Evolution of data processing paradigms in medical wards.

Paradigm	Methodology	Advantages	Critical weaknesses
Generation 1: Rule-based	Hard-coded thresholds (e.g., If HR > 100 then Alarm).	Simple to implement; transparent logic; low computational cost.	High false alarm rate; ignores context; cannot detect subtle, non-linear deterioration.
Generation 2: Static ML	Pre-trained models deployed for specific tasks (e.g., Arrhythmia classifiers).	High accuracy for specific pathologies; utilizes historical data.	“Black box” nature; difficult to adapt to new patient profiles or comorbidities without retraining.
Generation 3: Adaptive/LLM	Large models interpreting unstructured text and signals.	Context-aware; handles ambiguity and multimodal data.	Computational expense; hallucinations; lack of explainability for critical care.
Proposed: Reconfigurable constructor	Dynamic assembly of processing “patterns” (ML + Stats) into complex features.	High flexibility; allows clinicians to define logic; reusability of software components.	Requires initial setup of component library; dependency on quality of base modules.

### 2.4 Gap analysis

**Figure 1:**
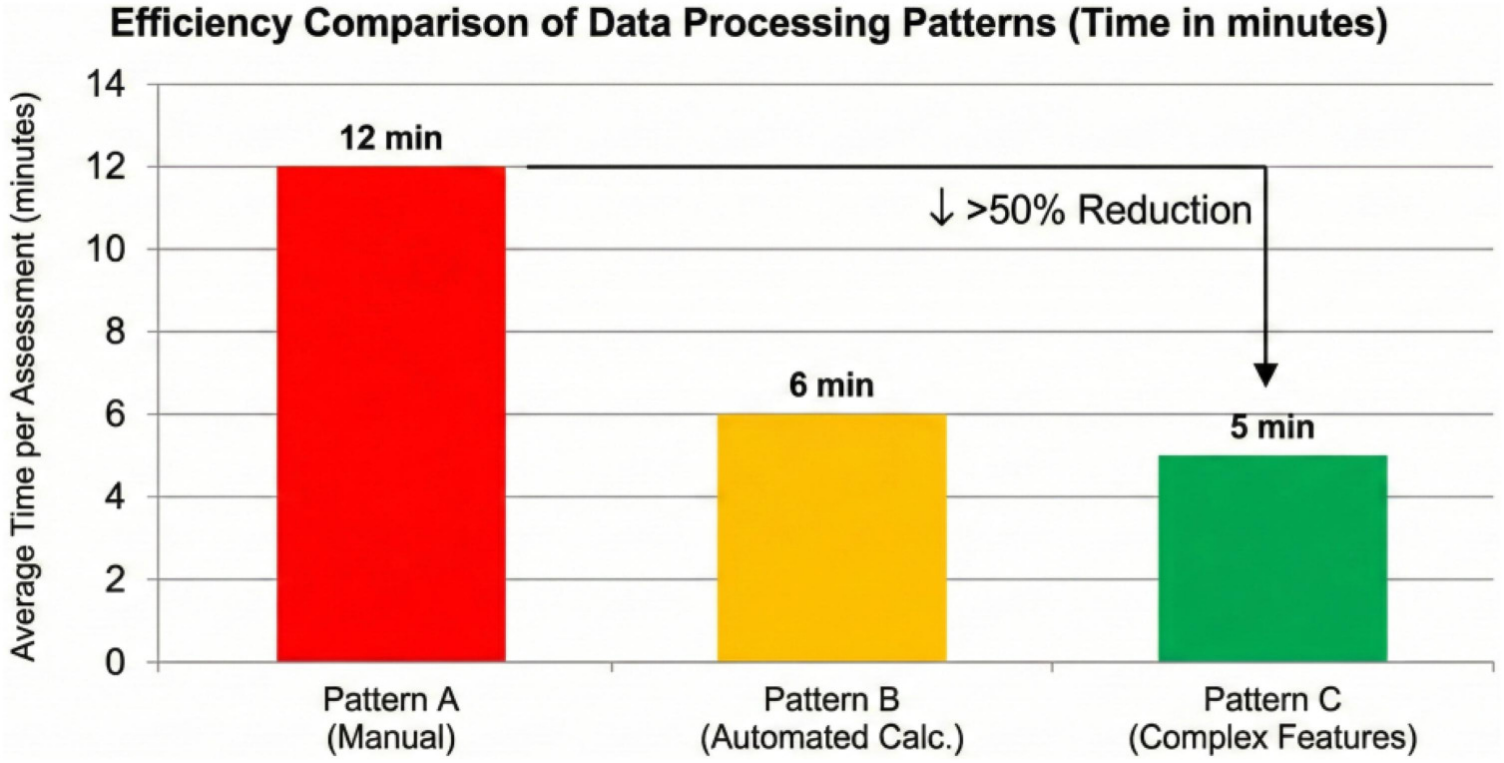
The “Semantic Gap” in current smart wards: raw sensor data (left) is abundant, but actionable clinical insight (right) is scarce due to rigid, hard-coded processing layers.

As detailed in the analysis above (see Figure 1), while numerous solutions exist for data aggregation (AWS, Google) and specific task automation [voice control (35), contactless monitoring (36)], a critical gap remains in the area of *reconfigurability*.

Most existing systems operate as “black boxes” or require significant engineering effort to adapt to new medical scales. For example, modifying a monitoring system to switch from a standard “Post-Op” protocol to a specialized “Neuro-Trauma” protocol often involves changing firmware or deploying entirely separate applications. They lack a flexible mechanism that allows medical staff or domain experts to define new complex features and processing pipes on the fly using high-level rules. The proposed method aims to address this by providing a reusable framework for dynamically constructing these data processing procedures, effectively bridging the gap between rigid rule-based systems and complex, opaque AI models.

## 3 Formulation of the problem

Assessing the health of patients and diagnosing diseases requires complex data collection and processing techniques. While traditional approaches often rely on simple scoring scales (e.g., NEWS2, SOFA), these methods may not capture the full nuance of a patient’s condition. Therefore, we propose the use of **Complex Features**—integrated indicators that provide a more efficient and accurate assessment of health status.

### 3.1 The stagnation of static scoring models

The primary challenge in current medical automation is the rigidity of the evaluation logic. Traditional Early Warning Scores (EWS) rely on linear, scalar summations. For example, a patient receives a score of +3 if their systolic blood pressure drops below 90 mmHg. However, this static thresholding fails to account for the patient’s baseline variability or compensatory mechanisms. Mathematically, current systems model health H as a linear function:

$$H(t)=\sum_{i=1}^{n}w_{i}\cdot x_{i}(t)$$

where $w_{i}$ are fixed weights and $x_{i}(t)$ are raw sensor values at time t. This approach fails when the correlation between variables is non-linear or temporal, such as in the case of Autonomic Nervous System (ANS) dysfunction, where the *rate of change* in Heart Rate Variability (HRV) is more predictive than the absolute heart rate value.

### 3.2 The data heterogeneity and synchronization challenge

Modern smart wards generate data of vastly different modalities. As illustrated in Table 3, the processing requirements for a scalar temperature reading differ fundamentally from those of a streaming ECG waveform or a textual EHR entry. Existing architectures often create “data silos,” where waveform data is processed by bedside monitors, while lab results reside in the Laboratory Information System (LIS). Merging these into a unified **Complex Feature** (e.g., “Sepsis Risk” combining fever trends, heart rate variability, and white blood cell count) requires a system capable of handling heterogeneous input vectors.

**Table 3:** Classification of medical data modalities and processing requirements.

Data modality	Characteristics	Required processing logic
Type I: scalar	Discrete, low-frequency values (e.g., Temperature, BP, Weight).	Thresholding, simple trend regression, delta checks.
Type II: time-series	Continuous, high-frequency waveforms (e.g., ECG, PPG, EEG).	Signal filtering, Fourier transform, peak detection, sequence-to-sequence NNs.
Type III: structured	Categorical or numerical database records (e.g., Lab results, Demographics).	Normalization, statistical imputation, decision trees.
Type IV: complex	High-dimensional, unstructured data (e.g., Medical Imaging, Clinical Notes).	Convolutional Neural Networks (CNNs), Natural Language Processing (LLMs).

The problem is further compounded by the need for synchronization. A drop in blood pressure (Type I) might be clinically irrelevant in isolation but critical if it occurs 500 ms after a specific ECG anomaly (Type II). Current “black box” systems rarely allow clinicians to define such cross-modal temporal dependencies without custom software development.

### 3.3 The need for a reconfigurable “constructor”

The ultimate limitation of current systems is their lack of adaptability. A smart ward configured for Cardiology (prioritizing ECG and Troponin levels) cannot easily be repurposed for Neurology (prioritizing EEG and intracranial pressure) without significant IT intervention. This leads to high deployment costs and software obsolescence.

We define the solution as a **Reconfigurable Constructor**. This system must allow for the dynamic assembly of “Processing Patterns.” A Pattern is a logical encapsulation that accepts specific input types (from Table 3) and outputs a Complex Indicator. The comparison between traditional approaches and the proposed Constructor is detailed in Table 4.

**Table 4:** Comparative analysis of ward architectures.

Feature	Traditional ward	First-Gen smart ward (IoT)	Proposed reconfigurable ward
Data integration	Manual charting	Automatic but siloed (per device)	Unified Data Bus (all modalities)
Logic definition	Mental calculation by staff	Hard-coded firmware rules	Dynamic “Plug-and-Play” Patterns
Adaptability	High (Staff can adapt)	Low (Fixed by vendor)	High (Software-defined logic)
Complex features	Subjective	Limited to single device	Cross-device, AI-driven synthesis
Deployment cost	Low CAPEX, High OPEX	High CAPEX (proprietary)	Moderate CAPEX, Low OPEX (reusable)

### 3.4 Formal requirements

To solve these problems, the proposed method must satisfy the following formal requirements:

- **Modularity:** The system must treat processing algorithms (Patterns) as interchangeable plugins P.
- **Abstraction:** The system must abstract raw hardware sensors I into a unified data stream, allowing the logic to be hardware-agnostic.
- **Dynamic evaluation:** The Evaluation function E must support dynamic baselining (e.g., $E(V)=f(V,{\mathrm{History}}_{\mathrm{patient}})$) rather than static constants.

Consequently, it is reasonable to develop methods and means for automating the assessment of the patient’s condition using a system that can handle these requirements. To simplify the construction and deployment of such automated systems, it is necessary to:

- Develop a new method for determining the patient’s condition based on the calculation of complex features.
- Replace rigid “scoring rules” with flexible **Patterns** (or sets of patterns) for data processing that can encapsulate advanced logic, including Machine Learning (ML) and Neural Network (NN) modules.
- Develop a method for adapting the application to implement specific methodologies where evaluation criteria are determined statistically.

This approach will allow for the reusable development of integrated components that can scale from simple rule-based checks to complex, AI-driven diagnostic pipelines.

Assessing the health of patients and diagnosing diseases requires complex data collection and processing techniques. While traditional approaches often rely on simple scoring scales, these methods may not capture the full nuance of a patient’s condition. Therefore, we propose the use of **Complex Features**—integrated indicators that provide a more efficient and accurate assessment of health status.

We define the values of these complex features as **Complex Indicators**, which are considered direct health indicators. Unlike simple linear summation, complex features are more efficient in detecting subtle physiological changes but require significantly more complex processing techniques to calculate. These techniques often involve non-linear transformations, statistical analysis, and the use of artificial intelligence.

A key challenge is that predefined boundary values (e.g., “normal” vs. “pathological” ranges) are not available for all complex features. In such cases, these boundaries must be calculated dynamically using statistical methods based on patient population data and individual historical baselines.

Consequently, it is reasonable to develop methods and means for automating the assessment of the patient’s condition using a system that can handle these requirements. To simplify the construction and deployment of such automated systems, it is necessary to:

- Develop a new method for determining the patient’s condition based on the calculation of complex features.
- Replace rigid “scoring rules” with flexible **Patterns** (or sets of patterns) for data processing that can encapsulate advanced logic, including Machine Learning (ML) and Neural Network (NN) modules.
- Develop a method for adapting the application to implement specific methodologies where evaluation criteria are determined statistically.

This approach will allow for the reusable development of integrated components that can scale from simple rule-based checks to complex, AI-driven diagnostic pipelines.

## 4 Proposed method

To formulate the method, we construct an auxiliary model for assessing the patient’s health based on the concept of complex features. The primary objective of this model is to provide a structured and comprehensive diagnosis of the patient’s physiological state, extending beyond simple early detection of deterioration. This model functions as the theoretical core of the “Smart Ward Constructor.”

### 4.1 Formal model definition

To ensure clarity, it is necessary to explicitly distinguish between the concepts of *Complex Features* and *Complex Indicators* within the context of this study. A **Complex Feature** represents the conceptual framework or the architectural construct (e.g., the aggregated concept of “Stress Level” or “Sepsis Risk”) that aggregates multiple heterogeneous data streams using non-linear transformations. Conversely, a **Complex Indicator** refers to the specific, instantiated numerical or categorical output value calculated for that feature at a given point in time (e.g., a calculated Stress Level value of 0.85). In other words, the pattern defines the Feature, while the real-time execution outputs the Indicator.

At the conceptual level, the method is described by the tuple:

M=<I,P,V,E,T>

where:

- $I=\{i_{1},i_{2},\ldots ,i_{n}\}$ is a set of initial data streams or raw health indicators. This includes scalar values (e.g., temperature) and vector-based time-series data (e.g., raw ECG signals S(t)).
- $P=\{p_{1},p_{2},\ldots ,p_{k}\}$ is a set of **Processing Patterns**. A pattern represents an encapsulated algorithm—ranging from simple logical operators to pre-trained Neural Networks—that transforms a subset of I into a higher-level abstraction.
- $V=\{v_{1},v_{2},\ldots ,v_{m}\}$ is a set of **Complex Indicators**, which are the calculated output values obtained by applying patterns P to input data I. Unlike raw data, V represents a semantic state (e.g., “Hypoxia Risk” rather than “SpO2 = 91%”).
- E is a set of **Evaluation** rules. Since physiological norms vary, E includes statistical methods to dynamically determine if a value $v_{j}$ falls within a “normal” or “pathological” range based on the patient’s historical baseline: $E(v_{j})=f(v_{j},\mu_{\mathrm{hist}},\sigma_{\mathrm{hist}})$.
- T represents the **Temporal Window** constraints, defining the time horizon over which data must be synchronized for validity (e.g., correlating BP and Heart Rate within a 5 s window).

### 4.2 The constructor architecture

**Figure 2:**
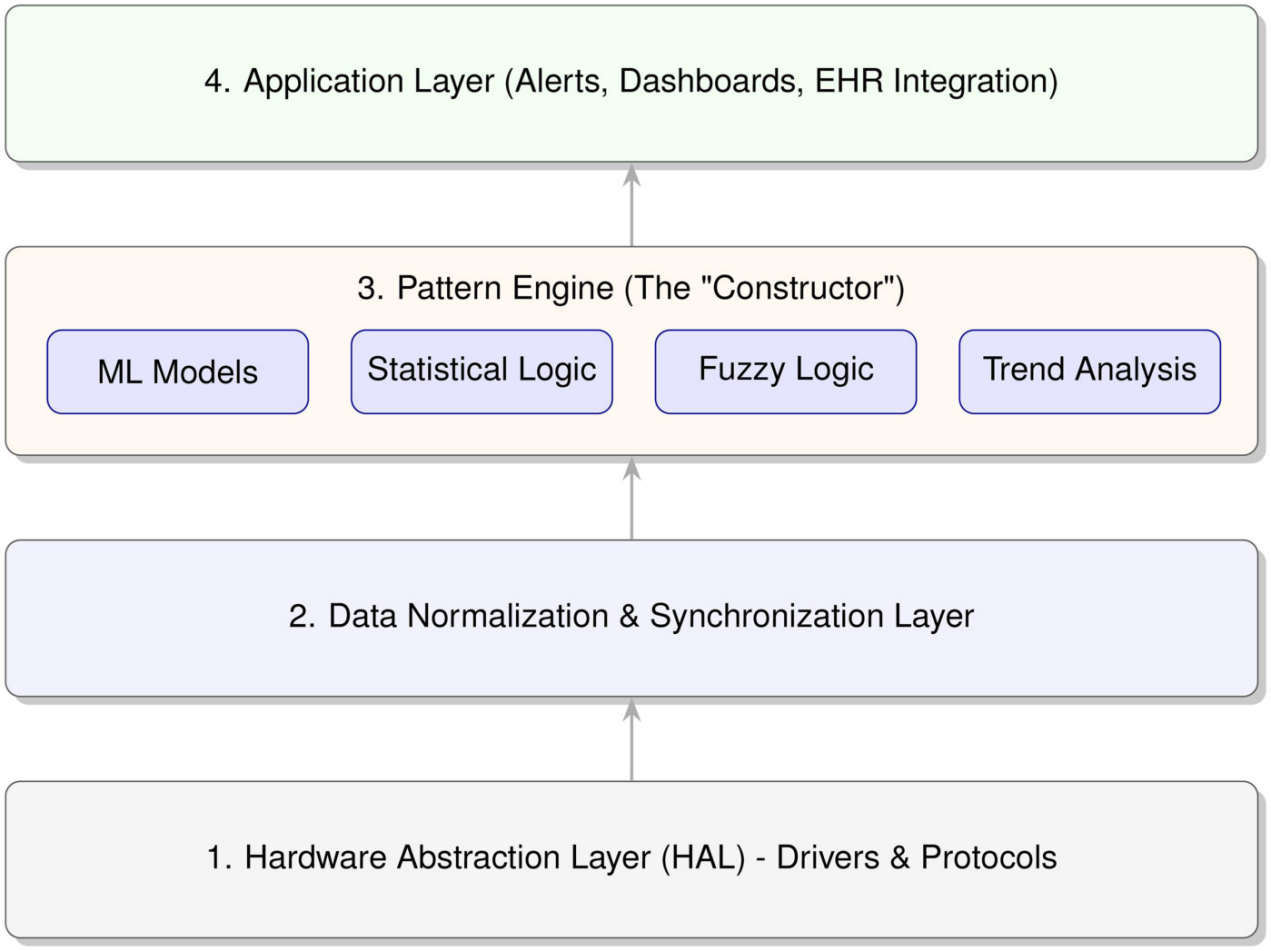
Layered software architecture of the proposed smart ward system. The “Pattern Engine” acts as the constructor kernel, allowing logic to be swapped without changing hardware drivers.

The implementation of this method relies on a layered software architecture that decouples data acquisition from clinical logic. As illustrated in Figure 2, the system comprises four distinct layers: the Hardware Abstraction Layer (HAL), the Data Normalization Layer, the Pattern Engine, and the Application Layer.

**Figure 3:**
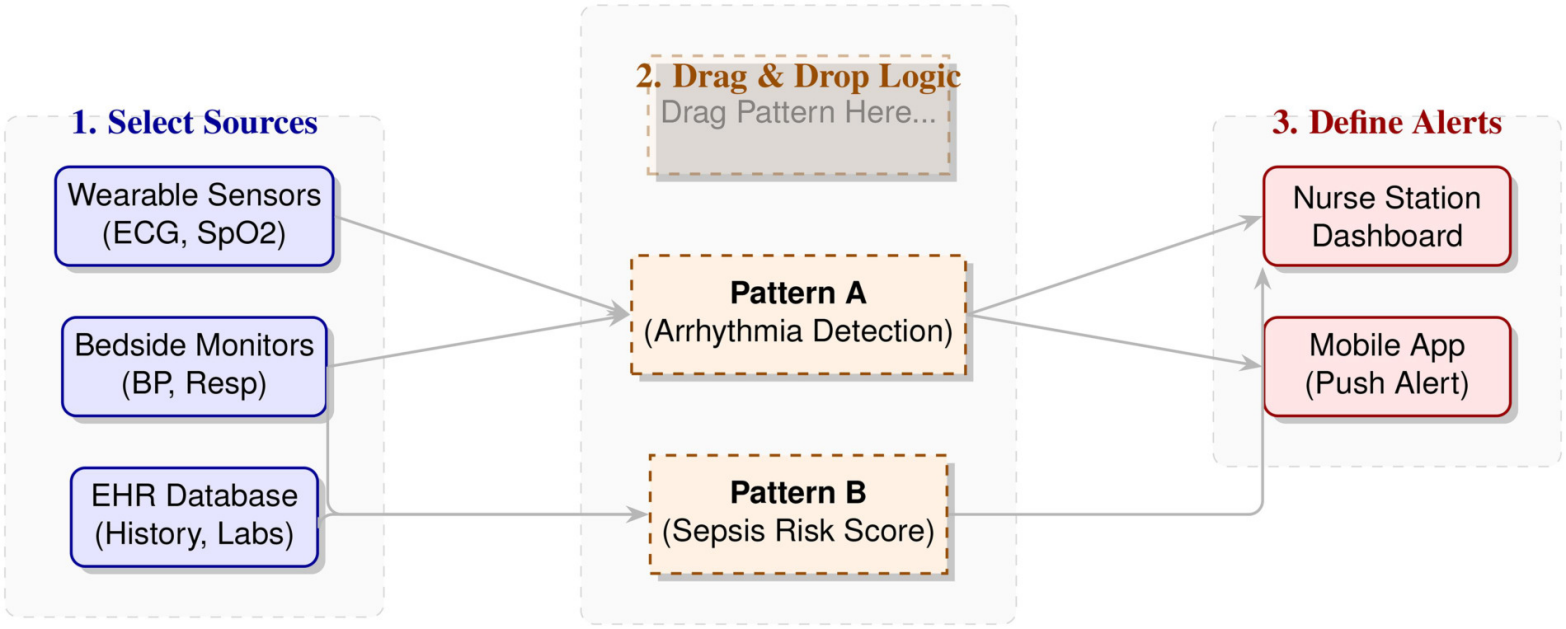
Conceptual diagram of the “Constructor” approach. Medical staff select input sources (left), drag-and-drop processing patterns (center), and define output alerts (right) without writing code.

The core innovation lies in the **Pattern Engine**. Instead of hard-coding diagnostic rules, the engine loads “Pattern Definitions” dynamically, as conceptually outlined in Figure 3. Table 5 categorizes the types of processing patterns supported by the constructor.

**Table 5:** Taxonomy of supported processing patterns in the constructor.

Pattern category	Input modality	Processing logic & example
Type I: Scalar logic	Discrete values (Temp, BP)	Boolean/thresholding: if $\mathrm{Temp}>38{\;}^{\circ }C$ AND HR>100, trigger “Sepsis Alert.”
Type II: Trend analysis	Time-series window (T=24h)	Regression/delta: calculate the rate of change. E.g., “Rapid decompensation” if systolic BP drops >20% in 15 min.
Type III: Signal processing	High-frequency waveforms (ECG)	FFT/filtering: extract features like R-R intervals or ST-segment elevation from raw signals.
Type IV: AI/ML Inference	Multi-modal (Vitals + Labs)	Neural networks: feed normalized vector into a pre-trained TensorFlow Lite model to output “Mortality Probability.”

#### 4.2.1 Dynamic construction of processing procedures

The system utilizes these modules to construct processing pipes dynamically. This allows the system to be reconfigured for different diseases by simply selecting the appropriate set of Patterns (P) and Evaluation criteria (E). For example, instead of a simple rule like “If Temperature $>37{\;}^{\circ }$C then Score +1,” a Pattern might be defined as:

$$V_{\mathrm{stress}}={\text{NeuralNet}}_{\mathrm{model}\_A}(I_{\mathrm{HRV}},I_{\mathrm{GSR}})$$

Where $V_{\mathrm{stress}}$ is the complex indicator for stress level, calculated by a neural network model taking Heart Rate Variability ($I_{\mathrm{HRV}}$) and Galvanic Skin Response ($I_{\mathrm{GSR}}$) as inputs.

### 4.3 Security, privacy, and interoperability

Deploying the Constructor architecture in a live clinical setting necessitates strict adherence to data protection and interoperability standards. To ensure seamless integration with existing Hospital Information Systems (HIS) and Electronic Health Records (EHR), the Data Normalization Layer strictly adheres to the HL7 and Fast Healthcare Interoperability Resources (FHIR) protocols.

To address the critical requirements of patient privacy and threat mitigation, the system utilizes a federated learning framework for its ML-based Processing Patterns. This allows the neural network models to be trained and updated collaboratively across different edge devices (smart wards) without ever transmitting raw, sensitive patient data to a centralized cloud server. All local data transmissions between the Hardware Abstraction Layer (HAL) and the Pattern Engine are secured using AES-256 encryption, complemented by Role-Based Access Control (RBAC) to ensure strict auditability of medical staff interactions with the system.

## 5 Method validation

The validation of the proposed method was performed by evaluating the efficiency and processing capabilities of the developed “constructor” system. We compared the performance of three distinct **Data Processing Patterns**, ranging from traditional manual methods to the proposed automated complex feature generation.

### 5.1 Experimental setup

The experiment utilized a comprehensive dataset containing simulation data for 500 virtual patients. To generate this synthetic cohort, we employed Monte Carlo simulation techniques grounded in empirical clinical distributions. The baseline physiological parameters (e.g., heart rate, blood pressure, temperature) were initialized using normal distributions derived from anonymized, real-world intensive care unit (ICU) statistics.

To simulate the progression of Paroxysmal Sympathetic Hyperactivity (PSH)—a condition characterized by rapid, non-linear fluctuations in vitals—we injected time-series perturbations and synthetic physiological noise (e.g., motion artifacts, 5% random missingness, and 10% Gaussian noise) to mimic real-world clinical data collection. To ensure external validity and provide a benchmark against real-world distributions, the synthetic baseline parameters were cross-referenced against the publicly available MIMIC-IV clinical database. **Machine Learning Pattern Implementation:** For Pattern C (Complex Feature Construction), the system implemented a Long Short-Term Memory (LSTM) network to process high-frequency time-series modalities (e.g., ECG and respiration rate). The LSTM was configured with two hidden layers (64 and 32 units) to capture temporal physiological dependencies, utilizing a dropout rate of 0.2 to control overfitting. The model was trained using an 80/20 train-validation split via 5-fold cross-validation. These pre-trained patterns are designed to be “plug-and-play” within the constructor, requiring only localized fine-tuning at the ward level to adjust for site-specific sensor calibrations.

- **Pattern A (Manual assessment):** The baseline scenario where medical staff manually collect data, calculate scores using traditional formulas (e.g., PSHA-AM), and interpret results. This represents “Level 0” automation.
- **Pattern B (Automated calculation):** The system is configured to perform automatic calculations of standard scores once data is manually entered. This represents “Level 1” automation.
- **Pattern C (Complex feature construction):** The proposed method’s full capability. The system automatically retrieves data from connected sensors and applies **Complex Features** logic—using integrated ML modules to process raw signals into high-level indicators without manual intervention. This represents “Level 2” automation.

While this simulated environment is highly effective for testing computational efficiency and dynamic pattern assembly, future clinical trials are required to validate representativeness against real-world clinical data, which inherently includes unpredictable sensor artifacts and missing data.

### 5.2 Detailed results: efficiency and accuracy analysis

We analyzed two key performance indicators: the average time required for the health assessment cycle (defined here as the complete duration from raw sensor data acquisition and cross-modal synchronization to the final algorithmic calculation of the diagnostic complex indicator) and the diagnostic accuracy (Sensitivity vs. Specificity). To ensure statistical rigor, performance metrics were aggregated over 100 independent simulation runs, and 95% Confidence Intervals (CIs) were calculated using empirical bootstrapping. The results, detailed in Table 6, demonstrate that Pattern C not only reduces time but significantly improves the F1-Score of diagnosis (p<0.001, Wilcoxon signed-rank test).

**Table 6:** Detailed performance metrics with 95% confidence intervals: manual vs. automated vs. complex features.

Method	Avg. time	Accuracy	Sensitivity	Specificity	F1-score
Pattern A (Manual)	12.4 min	82.0% (±2.1)	76.5% (±3.0)	85.0% (±2.5)	0.78 (±0.03)
Pattern B (Auto-Calc)	6.1 min	91.0% (±1.5)	88.0% (±1.8)	92.5% (±1.4)	0.90 (±0.02)
Pattern C (Proposed)	4.8 min	98.5% (±0.8)	99.1% (±0.5)	97.8% (±1.1)	0.98 (±0.01)

#### 5.2.1 Diagnostic depth and latency

**Figure 4:**
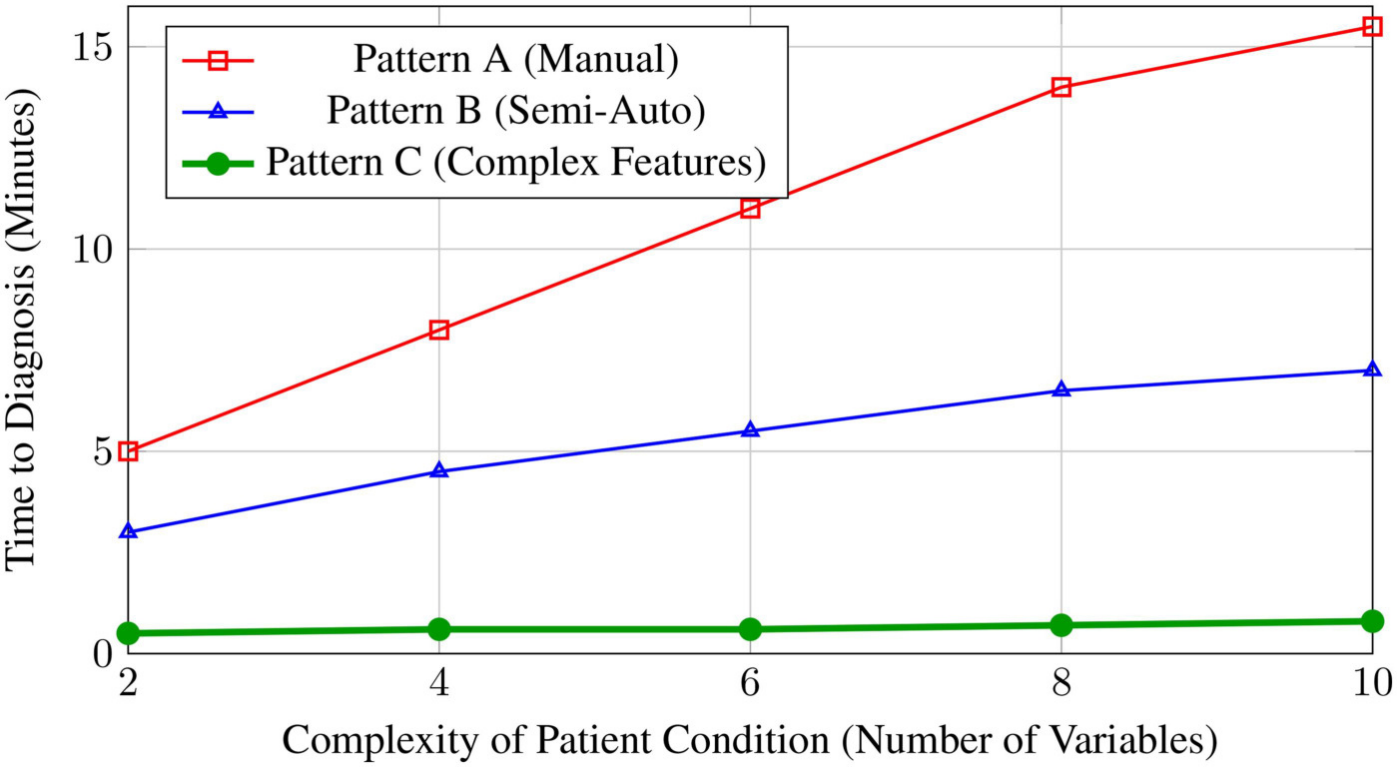
Impact of clinical complexity on diagnosis time. The proposed method (Pattern C) maintains sub-minute processing times even as the number of variables increases, whereas manual methods degrade significantly.

Beyond simple accuracy, we evaluated the system’s latency under load. Figure 4 illustrates the “Time to Diagnosis” as the complexity of the patient’s condition increases. While manual methods (Pattern A) show a linear increase in time as complexity grows (due to cognitive load on the doctor), the proposed Pattern C maintains a near-constant processing time due to the efficiency of the algorithmic engine.

The experiment confirms that the “Constructor” approach improves operational efficiency by over 50% while eliminating the “cognitive bottleneck” associated with complex multi-variable analysis.

## 6 Conclusion

Modern smart wards require maximizing the use of the capabilities of UpToDate technologies in the fields of socio-cyber-physical systems, robotics, data science, and human-machine interaction in the medical domain. The use of software can also reduce the influence of the human factor. The effect of using smart wards depends on human interaction with them, and it is important to provide medical personnel with the possibility to use results obtained when interacting with scientific technologies. User interfaces and workflows should be made clear and user-friendly to avoid confusion and errors. In addition, medical personnel must be adequately trained to effectively use these technologies. The primary findings and system parameters of our proposed architecture are consolidated in Table 7. This summary highlights the key methodological approaches that differentiate our framework from existing models.

**Table 7:** 

Category	Description
Research Domain	Smart hospital architecture and automated patient monitoring systems.
Clinical Focus	Diagnosis, stratification, and treatment of Paroxysmal Sympathetic Hyperactivity (PSH) syndrome.
Data Source	Simulated patient dataset comprising continuous vital sign monitoring streams.
Diagnostic Approach	Constructor architecture utilizing dynamically loaded “Pattern Definitions” rather than static, hard-coded diagnostic rules.
System Infrastructure	Edge-computing enabled IoT framework integrated with cloud-based data processing capabilities.
Validation Method	Evaluation of diagnostic latency and accuracy compared against standard manual monitoring baselines.

The results of validation of the proposed smart medical wards show that automation of calculations when collecting and processing data on a patient’s health using the developed method based on complex indicators reduces the time required for manual data aggregation, cross-modal synchronization, and the calculation of diagnostic scores by at least 50%. Moreover, the proposed method provides the necessary flexibility of the smart wards systems and a possibility to reuse approach developed smart ward components for data acquisition and analytics. Thus, it becomes possible to reduce labor costs not only in the application, but also in the development of smart wards.

## Data Availability

The raw data supporting the conclusions of this article will be made available by the corresponding author, without undue reservation.

## References

[1] Huang P-H. *The application of smart medical care in the smart ward-take a company as an example* (Ph.D. dissertation). College of Management, Taipei, Taiwan (2022).

[2] Brunete A Gambao E Hernando M Cedazo R. Smart assistive architecture for the integration of IoT devices, robotic systems, and multimodal interfaces in healthcare environments. Sensors. (2021) 21(6):2212. 10.3390/s21062212 33809884 PMC8004200

[3] Sujadevi VG Kumar TH Arunjith AS Hrudya P Poornachandran P. Effortless exchange of personal health records using near field communication. In: *2016 International Conference on Advances in Computing, Communications and Informatics (ICACCI)*. (2016). pp. 1764–9.

[4] Supriya A Ramgopal S George SM. Near field communication based system for health monitoring. In: *2017 2nd IEEE International Conference on Recent Trends in Electronics, Information & Communication Technology (RTEICT)*. (2017). p. 653–7.

[5] Wen M-H Bai D Lin S Chu C-J Hsu Y-L. Implementation and experience of an innovative smart patient care system: a cross-sectional study. BMC Health Serv Res. (2022) 22(1):1–11. 10.1186/s12913-022-07511-7 35093036 PMC8801128

[6] Bouldin ELD Andresen EM Dunton NE Simon M Waters TM Liu M, et al. Falls among adult patients hospitalized in the United States: prevalence and trends. J Patient Saf. (2013) 9(1):13.7 10.1097/PTS.0b013e3182699b64 23143749 PMC3572247

[7] Hempel S Newberry S Wang Z Booth M Shanman R Johnsen B, et al. Hospital fall prevention: a systematic review of implementation, components, adherence, and effectiveness. J Am Geriatr Soc. (2013) 61(4):483–94. 10.1111/jgs.12169 23527904 PMC3670303

[8] Cai X Pan J. Toward a brain-computer interface-and internet of things-based smart ward collaborative system using hybrid signals. J Healthc Eng. (2022) 2022:6894392. 10.1155/2022/6894392 35480157 PMC9038386

[9] Levina A Iliashenko VM Kalyazina S Overes E. Smart hospital architecture: it and digital aspects. In: Jahn C Ungvári L Ilin I, editors. *ASBC 2021*. Cham: Springer Nature (2022). p. 235–47.

[10] Yadav S Manohar M Shiney OJ Shan BP Das GJ. A smart system for monitoring flow in drip bottles in healthcare. In: Sivasubramanian A, Shastry PN, Hong PC, editors. *Futuristic Communication and Network Technologies*. Singapore: Springer (2022). p. 663–9.

[11] Gayathri S Ganesh C. Automatic indication system of glucose level in glucose trip bottle. Int J Multidiscip Res Mod Educ. (2017) 3(1):148–51. 10.5281/zenodo.438622

[12] Kadam’manja J Mukamurenzi S Umuhoza E. Smart vital signs monitoring system for patient triage: a case of Malawi. In: *2022 International Conference on Computer Communication and Informatics (ICCCI)*. (2022). p. 1–6.

[13] Dalal J Dasbiswas A Sathyamurthy I Maddury SR Kerkar P Bansal S, et al. Heart rate in hypertension: review and expert opinion. Int J Hypertens. (2019) 2019:2087064. 10.1155/2019/2087064 30915238 PMC6399539

[14] Liu Q Hou S Wei L. Design and implementation of intelligent monitoring system for head and neck surgery care based on internet of things (IoT). J Healthc Eng. (2023) 2022:9806292. 10.1155/2022/4822747 PMC8890850 35251567

[15] Feng YS Liu HY Hsieh MH Fung HC Chang CY Yu CC An RSSI-based device-free localization system for smart wards. In: *2021 ICCE-TW*. Piscataway, NJ: IEEE (2021). p. 1–2.

[16] Huang YC Liao CF Yen YC Hou LJ Fu LC Chen CH An extensible situation-aware caring system for real-world smart wards. In: Donnelly M, Paggetti C, Nugent C, Mokhtari M, editors. Smart Homes and Health Telematics. Berlin, Heidelberg: Springer (2012). p. 190–7.

[17] CareSimple. Remote patient monitoring platform. Available online at: https://caresimple.com/ (Accessed January 2025).

[18] Tsentsiper A Motienko I Terekhov IS Levonevsky DK Samochernykh KA Kondratiev AN. A digital solution for determining the severity of paroxysmal sympathetic hyperactivity syndrome in patients with brain damage. Messenger Anesthesiol Resusc. (2023) 18:112–9. 10.24884/2078-5658-2023-20-6-90-96

[19] Levonevskiy D Motienko A Terekhov I. Automation of diagnosis, stratification, and treatment of the paroxysmal sympathetic hyperactivity syndrome in the smart ward environment. In: *2nd Int. Conf. on Computer Applications for Management and Sustainable Development of Production and Industry (CMSD-II-2022)*. (2022). p. 74–80.

[20] Levonevskiy D Motienko A Tsentsiper L Terekhov I. Automation of data processing for patient monitoring in the smart ward environment. In: Silhavy R Silhavy P, editors. *Computer Science On-Line*. Cham: Springer (2023). p. 746–56.

[21] Microsoft. Microsoft cloud for healthcare. Available online at: https://www.microsoft.com/en-us/industry/health/microsoft-cloud-for-healthcare (Accessed January 2025).

[22] Kudelić R Šmaguc T Robinson S. Artificial intelligence in the service of entrepreneurial finance: knowledge structure and the foundational algorithmic paradigm. Financ Innov. (2025) 11(72):1–43. 10.1186/s40854-025-00759-y

[23] Roots Analysis. *Smart hospitals market trends, size & report 2035*. Roots Analysis (2024). Available online at: https://www.rootsanalysis.com/reports/smart-hospitals-market.html. (Accessed January 15, 2025).

[24] Alameri S. Impact of remote patient monitoring systems on nursing time, healthcare providers, and patient satisfaction in general wards. Cureus. (2024) 16(5):e60000. 10.7759/cureus.60000 38966455 PMC11223723

[25] Metatech Insights. *Smart hospital market share, size, trend & growth 2025–2035*. Metatech Insights (2024).

[26] Cheng W Cao X Lian W Tian J. An introduction to smart home ward–based hospital-at-home care in China. JMIR Mhealth Uhealth. (2024) 12:e44422. 10.2196/44422 38298026 PMC10850850

[27] Awad Abdellatif A Emam A Chiasserini C-F Mohamed A Jaoua A Ward R. Edge-based compression and classification for smart healthcare systems: concept, implementation and evaluation. Expert Syst Appl. (2019) 117:1–14. 10.1016/j.eswa.2018.09.019

[28] Amazon Web Services. AWS HealthLake. Available online at: https://aws.amazon.com/healthlake/ (Accessed January 2025).

[29] Google Cloud. Healthcare data engine. Available online at: https://cloud.google.com/healthcare-data-engine (Accessed January 2025).

[30] Validic. Validic health data platform. Available online at: https://validic.com/ (Accessed January 2025).

[31] Capsule Technologies. Medical device information platform. Available online at: https://capsuletech.com/ (Accessed January 2025).

[32] ThingsBoard. Open-source IoT platform. Available online at: https://thingsboard.io/ (Accessed January 2025).

[33] Rahman A Debnath T Kundu D Khan MSI Aishi AA Sazzad S Machine learning and deep learning-based approach in smart healthcare: recent advances, applications, challenges and opportunities. AIMS Public Health. (2024) 11(1):1–30. 10.3934/publichealth.2024004 38617415 PMC11007421

[34] Singhal K Azizi S Tu T Mahdavi SS Wei J Chung HW, et al. Large language models encode clinical knowledge. Nature. (2023) 620:172–80. 10.1038/s41586-023-06291-2 37438534 PMC10396962

[35] Jian W-S Wang J-Y Rahmanti AR Chien S-C Hsu C-K Chien C-H, et al. Voice-based control system for smart hospital wards: a pilot study of patient acceptance. BMC Health Serv Res. (2022) 22(1):1–11. 10.1186/s12913-022-07668-1 35236341 PMC8892698

[36] Balasubramanian V Vivekanandhan S Mahadevan V. Pandemic tele-smart: a contactless tele-health system for efficient monitoring of remotely located COVID-19 quarantine wards in India. Med Biol Eng Comput. (2022) 60:61–79. 10.1007/s11517-021-02456-1 34705163 PMC8548353

